# Healthcare consumption in congenital heart disease: A temporal life-course perspective following pediatric cases to adulthood

**DOI:** 10.1016/j.ijcchd.2023.100440

**Published:** 2023-01-11

**Authors:** Salma Pardhan, Zacharias Mandalenakis, Kok Wai Giang, Maria Fedchenko, Peter Eriksson, Mikael Dellborg

**Affiliations:** a Institute of Health and Care Sciences, University of Gothenburg, Sweden; b Department of Biomedical Sciences, KU Leuven, Belgium; c Adult Congenital Heart Unit, Sahlgrenska University Hospital, Gothenburg, Sweden; d Department of Molecular and Clinical Medicine, Sahlgrenska Academy, University of Gothenburg, Sweden; e Department of Medicine, Geriatrics and Emergency Medicine, Region Västra Götaland, Sahlgrenska University Hospital/Östra, Gothenburg, Sweden

**Keywords:** Congenital heart disease, Inpatient care, Life-course, Pediatric, Prevalence, Utilization

## Abstract

**Background:**

Improvements in diagnosis, intervention, and care of congenital heart disease (CHD) have led to increased survivability and lifelong dependence on healthcare. This study aims to determine the extensiveness of inpatient care episodes across different life-stages and CHD severity compared to matched controls, and to explore how healthcare utilization among pediatric CHD cases have changed over time.

**Methodology:**

National registry data was used to conduct a 1:9 matching analysis with age and sex matched controls. Then, Poisson timeseries analysis was used to conduct trend analysis for inpatient healthcare utilization among pediatric cases <18 years of age.

**Results:**

Most CHD cases were non-complex (87.3%), with highest hospitalization rates occurring in infancy. Mean number of hospitalizations among complex cases were over twice that of non-complex cases. Also, as age progressed, mean hospitalization for non-complex cases began converging to the control population. In terms of trend analysis within this study period, healthcare utilization increased by 34% among the infant categories, but decreased by 12% and 32% among children between 1-9 years and 10–17 years, respectively. Also, utilization was not trending in one direction substantiating the claim that multiple time periods are required to assess temporal changes within this population.

**Conclusion:**

Inpatient healthcare utilization among the CHD population appears to be decreasing over time in most cases, where non-complex cases transitioning to adult care are increasingly converging to the general population. Additionally, this study validates the need to use multiple time-periods when conducting longitudinal studies across the CHD population.

## 1 Introduction

Congenital Heart Disease (CHD) is an inborn structural heart defect, and is the most prevalent form of congenital anomaly [1,2], accounting for approximately one third of all major congenital malformations [1]. It occurs in roughly 1% of all live births [[2], [3], [4]], corresponding to approximately 1.35 million births per year worldwide [1]. Considerable improvements in diagnosis, intervention, and postoperative care have led to a substantial decrease in mortality among patients with CHD (5). Currently, over 97% of children born with CHD survive into adulthood [5], where majority are expected to have close to normal life expectancy [6].

CHD patients generally have a higher risk of mortality and morbidity than the general population [[7], [8], [9]], resulting in higher hospitalization and healthcare costs [10]. A recent American study found that hospital admissions between 2009 and 2013 increased more than 20% and 55% for patients with severe and non-severe CHD cases, respectively, leading to a 97.8% increase in inpatient costs [10]. However, there are inconsistencies in how the CHD population is analyzed over time. A number of studies have used two time-periods to assess temporal change [11,12], relying on the intuition that substantial innovative advancements in surgical practices and treatment for CHD led to increased survivability and reduced infant mortality, rendering these two time-periods as distinct [11]. However, there is no consensus on what these 2 time periods would be. Furthermore, studies exploring healthcare utilization among children and adolescents with CHD are limited [13].

The purpose of this paper is to gain a better perspective on how inpatient healthcare consumption has changed over the last few decades among CHD patients in Sweden, respective of disease severity. The two key objectives of this study are: 1) to determine the extent of inpatient healthcare consumption within Sweden across severe and non-severe cases in comparison to their matched non-CHD counterparts, and 2) to explore how healthcare utilization among children across different CHD severities have changed from 1970 to 2017. This study attempts to answer these questions using national Swedish registry data, where findings from this study will be instrumental in designing future temporal analysis for this population, as well as give much needed perspective on healthcare burden.

## 2 Methods

### 2.1 Definition of diagnosis

Diagnosis codes were used according to the International Statistical Classification of Diseases and related Health Problems (ICD) system: ICD-8 (1968–1986), ICD-9 (1987–1996), and ICD-10 (1997-onwards) (Supplementary Table 1). CHD was defined as any patient with at least one outpatient visit, a hospital discharge, or a death certificate with a registered diagnosis of CHD.

The CHD diagnoses were grouped into six lesion groups according to a hierarchical classification system based on lesion severity, originally presented by Botto et al. [14] as modified by Liu et al. [15]. Complex cases were defined as cases in lesion groups 1 or 2, and non-complex cases were defined as cases within lesion groups 3–6 (Supplementary Table 2). In general terms, complex CHD cases were defined as patients with conotruncal or severe non-conotruncal defects. Non-complex cases were defined as patients with coarctation of the aorta, ventricular septal defect, atrial septal defect, and all other defects including valvular defects. Healthcare utilization was qualified as total number of hospitalizations within each age category.

### 2.2 Study population and statistical analysis

This study includes all live-born infants born in Sweden with a registered CHD diagnosis between January 1, 1970 and December 31, 2017. Analysis for this study were based on Swedish national registry data, which has full coverage and mandatory participation. In particular, data on patients with registered diagnosis of CHD were provided by 1) the Swedish National Patient Register (NPR), which collects data on both primary and secondary discharge diagnoses and all medical procedures for all in-hospital patients [14], and 2) the Cause of Death Register (CDR), which collects mortality data including cause of death and contributing factors [16].

The Total Population Register (TPR), which collects demographic data from 1968 onwards [17], was used to match cases with controls. Previous studies demonstrated high validity of the NPR and CDR for cardiovascular diseases [18,19], where the NPR has a positive predictive value of 85%–95% [18]. Data across registries were merged using national Personal Identity Numbers, which all Swedish residents obtain at either birth or upon migration into the country.

Due to dataset limitations, this retrospective study is limited to individuals born in Sweden <47 years, where the trend analysis is limited to pediatric cases <18 years of age. No other exclusions were made. Healthcare utilization was defined as number of inpatient visits and were broken into 5-year intervals in an effort to adequately analyze consumption trends. All statistical analyses were performed using R software (R Core Team (2022). R: A language and environment for statistical computing. R Foundation for Statistical Computing, Vienna, Austria. URL https://www.R-project.org/).

#### Case-control utilization comparison using attribute matching

The case population included all CHD patients regardless of the presence of syndromes. Attribute matching, which evaluates the Average Treatment Effect of the Treated, was used due to the small number of confounders and the large number of available controls. The first part of this study matched CHD patients by birth year and sex with 10 random controls free of CHD, using the TPR. Due to the exclusion criteria of being born in Sweden between 1970 and 2017, a total of 8.61 controls were available for each CHD patient. The time intervals, which are based on the follow-up time from birth, reflect the various life-course of an individual: infants under the age of 1, young children aged 1–9 years, older pediatric cases aged 10–17 years, young adults aged 18–29, and adults aged 30–39 and 40+ years. Only individuals that survive into the succeeding time interval were included in that interval.

#### Trend analysis using Poisson regression

Time series analysis were performed based on Poisson regression models to estimate trends across severe and non-severe cases for inpatient healthcare utilization across the following pediatric birth cohorts: 1) up to first year of life, 2) 1 to <10 years, and 3) 10 to <18 years. Cohort categories were devised based on the rationale that the most extensive interventions are introduced in the first year of life, followed by the first 10 years of life, and gradually reduces thereafter. Poisson regressions were employed as healthcare utilization was defined as number of hospitalizations. The regressed population was limited to individuals <18 years of age, as complete follow-ups were only available for these age groups.

The reference period for the regression analysis was set to 1970–1974, as this was the first time-interval in the dataset. Each 5-year interval of time was analyzed and compared in order to ascertain how much inpatient healthcare utilization had changed overtime. Statistical significance (p ≤ 0.05) was interpreted as significant change in trend. Output was reported as incidence rates, where exponentiated coefficients were compared to the reference period.

No overdispersion is assumed as the distribution variance and mean were similar. However, Quasi-Poisson regressions were also conducted in the event of overdispersion (Supplementary Tables 3–5), where results were very similar, and issues of significance were mainly detected in complex cases between the 10 – <18-year age category.

Since hospitalization was calculated as registered inpatient visits, misclassification may occur when some patients are registered as new admissions when moved from one hospital or care unit to another. Different hospitals and patient care units have different policies for coding admissions, which are neither transparent nor consistent. As a sensitivity measure, we explored the number of pediatric hospitalizations across the three different age intervals to determine if there were extreme utilizations that could be due to these intrinsic administrative data limitations (Supplementary Table 6). Across the three included age categories, 99.6%, 98.9%, and 99.43% of infant, “young pediatric”, and “older pediatric” cases had 0-14 hospitalizations, respectively. Thus, we considered >14 hospitalizations to be extreme cases and reran the Poisson regressions excluding these cases. Results of this sensitivity analysis did not yield findings that were drastically different from the main regressions (Supplementary Table 7).

### 2.3 Ethical approval

Ethics approval for this study was granted from the Gothenburg Regional Research Ethics Board (approval numbers 912–16 and T 619–18) which complies with the Declaration of Helsinki. The need for informed consent was waived by the Ethics board as all registries used in this study are government administered and have obtained ethics approval [20,21]. Anonymity of subjects were established by replacing national registration numbers with code in the final data set, as set by the Swedish National Board of Health and Welfare.

## 3 Results

### 3.1 Case-control matching analysis

The present study identified 67,814 patients diagnosed with any form of CHD (50.4% male) between January 1, 1970, and December 31, 2017. The study population was based on CHD patients and matched controls born in Sweden between 1970 and 2017. Descriptive statistics for matched baseline data are presented as frequencies and percentages in Table 1. The mean birth year for the CHD population was 1999.6 ± 12.8 where majority of CHD births are post 2000. Most of the CHD population are non-complex cases (87.3%). The prevalence of CHD began to rise from the mid 1980s, where most of the population (81.1%) were pediatric cases under the age of 18. The highest concentration of CHD cases are within the infant age category (<1 years old) and slowly decline in proceeding age categories thereafter.

**Table 1:** Characteristics of the study population.

	Case	Controls
	(N = 67,814)	(N = 583,709)
Gender		
Female	33,641 (49.6%)	289,563 (49.6%)
Male	34,173 (50.4%)	294,146 (50.4%)
Mean birth year	1999.6 ± 12.8	2000.6 ± 12.6
Median birth year [interquartile range]	2003 [1990; 2010]	2004 [1992; 2011]
By birth period, n (%)		
1970–1974	3397 (5.0%)	26,812 (4.6%)
1975–1979	3363 (5.0%)	25,031 (4.3%)
1980–1984	3749 (5.5%)	26,625 (4.6%)
1985–1989	5184 (7.6%)	38,629 (6.6%)
1990–1994	7027 (10.4%)	58,546 (10.0%)
1995–1999	6027 (8.9%)	51,318 (8.8%)
2000–2004	9114 (13.4%)	79,941 (13.7%)
2005–2009	11,258 (16.6%)	100,689 (17.2%)
2010–2014	11,864 (17.5%)	109,973 (18.8%)
2015–2017	6831 (10.1%)	6,6145 (11.3%)
By age intervals, n (%)		
0 - < 1 Years	67,814 (32.3%)	583,709 (31.1%)
1 - < 10 Years	62,050 (29.6%)	562,779 (30.0%)
10 - < 18 Years	40,345 (19.2%)	364,461 (19.4%)
18 - < 30 Years	24,774 (11.8%)	225,215 (12.0%)
30 - < 40 Years	10,727 (5.1%)	97,537 (5.2%)
40+ Years	4172 (2.0%)	41,260 (2.2%)
Mean Population (%) by Lesion Complexity^a^		
Complex CHD	8640 (12.7%)	72,093 (12.4%)
Non-complex CHD	59,174 (87.3%)	511,616 (87.6%)

a Lesion complexity defined in Supplementary Table 2.

**Fig. 1:**
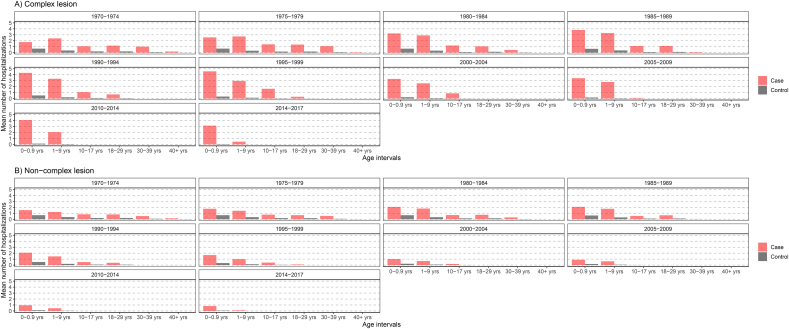
Mean number of hospitalizations for case and controls, by severity and birth period.

**Fig. 2:**
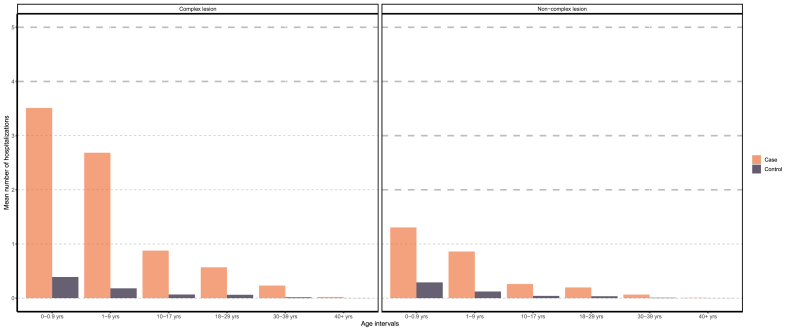
Mean hospitalization for case and controls, by severity and age category.

Most hospitalizations occurred at infancy and gradually declined thereafter, where mean hospitalization among complex CHD cases were over twice that of non-complex cases (Fig. 1, Fig. 2). However, we find that as age increases mean hospitalization for non-complex cases converge to the general population. In particular, after 1995, health consumption between controls and non-complex cases over the age of 18 are arguably the same.

### 3.2 Trend analysis

**Fig. 3:**
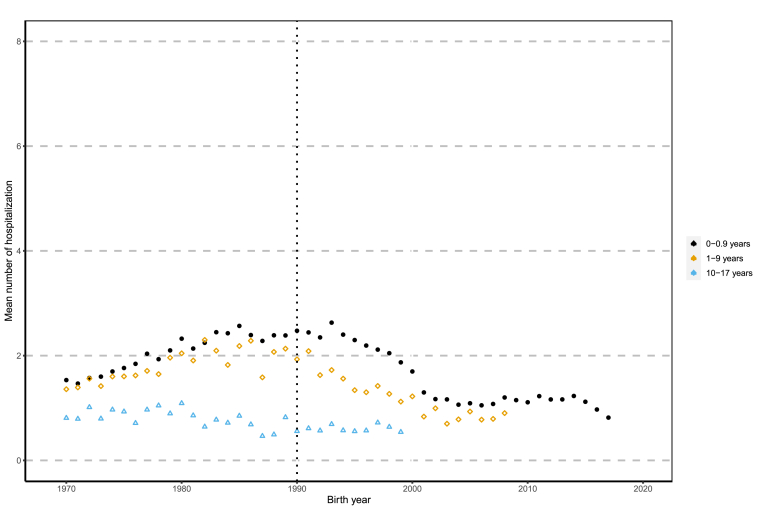
Time trend of hospitalization for all pediatric CHD cases.

Overall, relative changes in hospitalizations across all CHD severities were significantly different from the reference period (1970–1974) for all birth periods except for “older pediatric cases” between 1975 and 1979 (Table 2). When exploring this age category by severity (Table 3, Table 4), hospitalization in all but complex cases were significantly different from the base year between 1985 and 1999. Hospitalization among infants, regardless of severity, were significantly higher than the base period, though only the complex cases increased steadily across time. Utilization among non-complex infant cases peaked between 1980 and 1984 and began decreasing by 1995–1999. Similar pattern of utilization can be seen for non-complex “young pediatric cases”, where utilization falls 18% below 1970–1974 levels. The most drastic decrease in utilization is observed among the “older pediatric cases” (10 to < 18 years), where hospitalization was 49% less than 1970–1974 values among non-complex cases. Complex cases in this age category, however, remained higher than 1970–1974 values, where hospitalization was 52% higher in 1995–1999 compared to the base period. Hospitalizations across all age groups and severities are illustrated in Fig. 3 and Supplementary Fig. 1.

**Table 2:** Poisson Regression Results on Changes in Hospitalization Among Total CHD Population Over Time.

	Infant Cases: Age interval (0 - < 1 years)		Young Pediatric Cases: Age interval (1 - < 10 years)		Older Pediatric Cases: Age interval (10 - < 18 years)	
**Birth Period**	Relative change in hospitalization	Pr (>|z|)	Relative change in hospitalization	Pr (>|z|)	Relative change in hospitalization	Pr (>|z|)
1970–1974	***REFERENCE YEAR***					
1975–1979	1.23	0.00	1.16	0.00	1.03	0.25^a^
1980–1984	1.47	0.00	1.38	0.00	0.92	0.00
1985–1989	1.52	0.00	1.38	0.00	0.74	0.00
1990–1994	1.56	0.00	1.21	0.00	0.68	0.00
1995–1999	1.34	0.00	0.88	0.00	0.68	0.00

^a^ significant to 5%

^b^ significant to 10%

^c^ insignificant

**Table 3:** Poisson Regression Results on Changes in Hospitalization Among Complex CHD Cases Over Time.

	Infant Cases: Age interval (0 - < 1 years)		Young Pediatric Cases: Age interval (1 - < 10 years)		Older Pediatric Cases: Age interval (10 - < 18 years)	
**Birth Period**	Relative change in hospitalization	Pr (>|z|)	Relative change in hospitalization	Pr (>|z|)	Relative change in hospitalization	Pr (>|z|)
1970–1974	***REFERENCE YEAR***					
1975–1979	1.45	0.00	1.14	0.00	1.3	0.00
1980–1984	1.82	0.00	1.21	0.00	1.12	0.02^a^
1985–1989	2.15	0.00	1.37	0.00	1.03	0.48^c^
1990–1994	2.43	0.00	1.39	0.00	0.99	0.84^c^
1995–1999	2.58	0.00	1.24	0.00	1.52	0.00

^a^ significant to 5%

^b^ significant to 10%

^c^ insignificant

**Table 4:** Poisson Regression Results on Changes in Hospitalization Among Non-Complex CHD Cases Over Time.

**Birth Period**	**Infant Cases:** **Age interval (0 - < 1 years)**		**Young Pediatric Cases:** **Age interval (1 - < 10 years)**		**Older Pediatric Cases:** **Age interval (10 - < 18 years)**	
	**Relative change in hospitalization**	**Pr (>|z|)**	**Relative change in hospitalization**	**Pr (>|z|)**	**Relative change in hospitalization**	**Pr (>|z|)**
1970–1974	**REFERENCE YEAR**					
1975–1979	1.15	0.00	1.17	0.00	0.93	0.02^a^
1980–1984	1.36	0.00	1.47	0.00	0.84	0.00
1985–1989	1.36	0.00	1.44	0.00	0.66	0.00
1990–1994	1.36	0.00	1.2	0.00	0.61	0.00
1995–1999	1.1	0.00	0.82	0.00	0.51	0.00

^a^ significant to 5%

^b^ significant to 10%

^c^ insignificant

## 4 Discussion

Timely discussions on utilization trends are necessary to begin understanding resource implications for specialized care, especially considering the increasing prevalence of non-complex cases of CHD. To our knowledge, this is the first case-control study to provide a life-course perspective on healthcare utilization, and the first study to evaluate temporal trends across the pediatric CHD population with 17 full years of follow-up time within a universal healthcare framework. Principle findings include: 1) the majority of Swedish CHD cases are non-complex, where inpatient healthcare utilization among this group is converging to utilization amounts of the general non-CHD population around the time these patients transition to adult care; and 2) inpatient healthcare consumptions among CHD patients are not only trending upwards. Rather, it had increased for a period of time, and is now decreasing within the Swedish pediatric population, which is most evident in non-complex cases. This will have consequential impact on discussions pertaining to healthcare sustainability and burden for this population.

When exploring Fig. 3 and Supplementary Fig. 1, this shift in healthcare utilization becomes apparent. If one was to insert a best fit line to these figures, it is evident that over-estimation of utilization in the latter years would occur for most age-groups and severities. This is most obvious in non-complex cases, across all age categories. However, the birth period at which point this change in trend occurs is not clear from this analysis and is different for each age category and level of complexity.

There are several possible explanations for these varying trends across age groups and severity. Firstly, the late 1950's to the late 1970's heralded several novel operative techniques, such as the Mustard and Senning operations followed by the Jatene procedure for treating transposition of the great arteries or the introduction of the Fontan procedure for tricuspid atresia palliation [22], with a possible learning curve from initial introduction to successful and appropriate adoption and application of these techniques. Secondly, the ability to maintain the ductus arteriosus patent provided important time for diagnosis and hitherto not available techniques [22]. Thirdly, diagnostic cardiac catheterization became unnecessary for most non-complex cases given the introduction of echocardiography [23]. Together, these advances meant that more children with complex heart diseases were operated, fewer were hospitalized for diagnostic catheterization, and more procedures were completed at an earlier age. Advances in intrauterine diagnosis can be argued to have changed the spectrum of children born with severe heart malformation resulting in termination of pregnancy. However, such terminations are expected to have minimal to negligible impact on overall health consumption as such terminations are comparatively few. Sufficed to say, all these changes may have very different effects in the respective category of congenital heart defects but the overall effect for the CHD population as a whole is lower consumption of health care in later years, and with age.

### Study strengths

This study provides a novel life-course perspective on healthcare utilization over a long timespan where the case population is compared to a large matched non-CHD population. This substantially contributes to the robustness of this study. This research also provides meaningful temporal analysis among the pediatric CHD population, where all patients were fully followed-up for 17 years. Previous utilization studies were limited as they either used short timeframes (less than 12 years) [6,10,13,[24], [25], [26]], did not focus on specialized cardiac care [6,27], and/or did not compare utilization with the general population. Additionally, this study's findings on hospitalization rates across different age categories are aligned with a recent American study exploring healthcare utilization among adolescents with CHD (13).

Also, this research draws its strengths from Sweden's large and high-quality patient registries where access to healthcare is not strikingly compromised or limited due to location or personal income.

### Study limitations

The restriction of analysis to only inpatient care, as the Swedish outpatient registry data was only available from 2000 onwards, along with the inability to extend the analysis to different forms of inpatient care form the key limitations of this study. Also, the study population is relatively young, where less than 5% of the case-population was over 40 years of age and the oldest patient included was 46 years old. Furthermore, although all registries used were previously validated, there is an intrinsic vulnerability in all registry-based retrospective studies for misclassification due to human error in coding data, residual confounders, and selection bias. The data is also somewhat vulnerable to overreporting inpatient visits as inter- and intra-hospital transfers may be coded as unique hospital visits. However, as per the conducted sensitivity analysis, this is likely to have insignificant implications on the overall analysis where both cases and controls face similar realities with regards to overreporting inpatient visits. Thus, the difference between cases and controls for overreporting are likely negligible.

The outcome variable in this study was defined as number of inpatient hospital admissions as opposed to length of hospital stay. We argue that quantifying the number of hospital visits more accurately reflects number of ailments, issues, and complications due to CHD without sullying the results with length of hospital stay policies. Arguably, limited interpretability and applicability to cost studies is the obvious trade-off. Furthermore, number of inpatient hospital admissions do not differentiate between unique patient care episodes and inter- and intra-hospital transfers and follow-up care for both cases and controls. However, considering the large case and control population used in this study, this reality will have minimal impact on study results.

### Future area of research

In order to validate the generalizability of this study as well as to create meaningful analysis of burden on healthcare, further international studies across different healthcare systems and levels of care focusing on health consumption trends and costs within the CHD population is necessary. Of particular interest would be costs studies exploring burden on healthcare across lesion groups to address concerns regarding long-term sustainability of care. Extension of analysis to outpatient care would be greatly beneficial, especially among the adolescent population who mainly encounter care in the form of outpatient visits [13]. Considering there are various levels of CHD severities where CHD patients have higher risks of mortality and morbidity compared to the general population [[7], [8], [9]], investigation on different forms of intervention and non-cardiac related healthcare utilization, and its subsequent burden on the healthcare system, is also warranted. Lastly, although this study supports the claim that temporal analysis for this population should occur across at least two different time periods, more investigation is needed as to when these distinct time periods are, and if they vary across CHD severity or population age cohort groups.

## Conclusion

This research explores inpatient healthcare utilization over time across the CHD population which is crucial to the discussion on future healthcare sustainability. This study supports the assertion that non-complex cases of CHD are outpacing complex cases, where the largest burden on healthcare occurs in infancy. The key findings of this study are that inpatient utilization among the CHD population appears to be decreasing over time in most cases, where non-complex cases transitioning to adult care are increasingly converging to the general population. Additionally, this study validates the need to use multiple time-periods when conducting longitudinal studies across the CHD population, as simple time-series analysis could overestimate healthcare utilization. However, further research is needed to determine what these time periods are.

## Declaration of competing interest

The authors declare that they have no known competing financial interests or personal relationships that could have appeared to influence the work reported in this paper.
