# Supplementary material for: Healthcare consumption in congenital heart disease: A temporal life-course perspective following pediatric cases to adulthood
Source: Int J Cardiol Congenit Heart Dis. 2023 Jan 11;11:100440. doi: 10.1016/j.ijcchd.2023.100440 (PMC11657615; doi:10.1016/j.ijcchd.2023.100440)
Supplement: Multimedia component 2 [file mmc2.docx]

**Supplementary Table 1: ICD Codes**

| Supplemental Table 1: ICD 8,9 and 10 diagnose codes for congenital heart disease. | | | |
| --- | --- | --- | --- |
| Diagnosis | **ICD 8** | **ICD 9** | **ICD 10** |
| Transposition of the great arteries | 746,19 | 745B | Q203 |
| Common arterial trunk | 746,09 | 745A | Q200 |
| Tetralogy of Fallot | 746,29 | 745C | Q213 |
| Ventricular septal defect | 746,39 | 745E | Q210 |
| Atrial septal defect/patent foramen ovale | 746,42 | 745F | Q211 |
| Congenital tricuspid stenosis or atresia | 746,54 | 746B | Q224 |
| Ebstein’s anomaly | 746,54 | 746C | Q225 |
| Congenital stenosis of the aortic valve | 746,73 | 746D | Q230 |
| Congenital insufficiency of the aortic valve | 746,79 | 746E | Q231 |
| Congenital mitral stenosis | 746,59 | 746F | Q232 |
| Congenital mitral insufficiency | 746,59 | 746G | Q233 |
| Hypoplastic left heart syndrome | 746,74 | 746H | Q234 |
| Congenital subaortic stenosis | 746,79 | 746W | Q244 |
| Cor triatriatum | 746,89 | 746W | Q242 |
| Infundibular pulmonic stenosis | 746,63 | 746W | Q243 |
| Congenital coronary artery anomalies | 747,69 | 746W | Q245 |
| Congenital heart block | 746,89 | 746W | Q246 |
| Coarctation of the aorta | 747,19 | 747B | Q251 |
| Interruption of the aortic arch | 747,19 | 747B | Q252  Q253 |
| Congenital malformations of the pulmonary artery | 747,34  747,39 | 747D | Q255  Q256  Q257 |
| Congenital malformations of the great veins | 747,49  747,59 | 747E | Q260  Q261  Q262  Q263  Q264 |
| Cor biloculare | 746,89 | 745H | Q208 |
| Double outlet right ventricle | 746,19 | 745B | Q201 |
| Double outlet left ventricle | 746,19 | 745B | Q202 |
| Double inlet ventricle | 746,37 | 745D | Q204 |
| Congenitally corrected transposition/discordant atrioventricular and ventriculoatrial connection | 746,19 | 745B | Q205 |
| Isomerism of atrial appendages | 746,89 | 745W | Q206 |
| Unspecified congenital malformations of the cardiac chambers | 746,89 | 746X | Q208  Q209 |
| Atrioventricular septal defect | 746,47  746,46  746,43 | 745G | Q212 |
| Aortopulmonary septum defect | 746,09 | 745A | Q214 |
| Unspecified congenital malformations of the cardiac septum | 746,99 | 745X | Q219 |
| Pulmonary valve atresia | 746,64 | 746A | Q220 |
| Congenital stenosis of the pulmonary valve | 746,63 | 746A | Q221 |
| Congenital pulmonary valve insufficiency | 746,69 | 746A | Q222 |
| Hypoplastic right heart syndrome | 746,69 | 746B | Q226 |
| Patent ductus arteriosus | 747,09 | 747A | Q250 |
| Other congenital malformations of the cardiac septum | 746,89 | 745W | Q218 |
| Other unspecified congenital malformations of the aorta | 747,29 | 747C | Q254  Q258  Q259 |
| Other congenital malformations of the pulmonary valve | 746,69 | 746A | Q223 |
| Other congenital malformations of the tricuspid valve | 746,54 | 746B | Q228  Q229 |
| Other congenital malformations of aortic and mitral valves | 746,89 | 746W | Q238  Q239 |
| Other specified congenital malformations of the heart | 746,89 | 746W | Q248 |
| Unspecified congenital malformations of the heart | 746,99 | 746X | Q249 |
| ICD=International Statistical Classification of Diseases | | | |
