# Supplementary material for: Healthcare consumption in congenital heart disease: A temporal life-course perspective following pediatric cases to adulthood
Source: Int J Cardiol Congenit Heart Dis. 2023 Jan 11;11:100440. doi: 10.1016/j.ijcchd.2023.100440 (PMC11657615; doi:10.1016/j.ijcchd.2023.100440)
Supplement: Multimedia component 3 [file mmc3.docx]

**Supplementary Table 2: CHD diagnosis constituting 6 CHD Lesion groups, corresponding to ICD 8-10 versions**

| The congenital heart disease diagnoses that constitute the six congenital heart disease lesion groups and their corresponding diagnoses in ICD 8, 9 and 10 versions. | | | | |
| --- | --- | --- | --- | --- |
| **Lesion group** | **CHD diagnosis** | **ICD 8** | **ICD 9** | **ICD 10** |
| Lesion group 1 | Double outlet left ventricle | 746.19 | 745B | Q202 |
|  | Transposition of great arteries | 746.19 | 745B | Q203 |
|  | Congenitally corrected transposition/discordant atrioventricular and ventriculoatrial connection | 746.19 | 745B | Q205 |
|  | Tetralogy of Fallot | 746.29 | 745C | Q213 |
|  | Common arterial trunk | 746.09 | 745A | Q200 |
|  | Aortopulmonary septum defect | 746.09 | 745A | Q214 |
|  | Double outlet right ventricle | 746.19 | 745B | Q201 |
| Lesion group 2 | Hypoplastic left heart syndrome | 746.74 | 746H | Q234 |
|  | Endocardial cushion defect/ atrioventricular septal defect | 746.43 746.46 746.47 | 745G | Q212 |
|  | Common ventricle | 746.37 | 745D | Q204 |
| Lesion group 3 | Coarctation of the aorta | 747.19 | 747B | Q251 |
| Lesion group 4 | Ventricular septal defect | 746.39 | 745E | Q210 |
| Lesion group 5 | Atrial septal defect | 746.42 | 745F | Q211 |
| Lesion group 6 | All other congenital heart disease diagnoses that are not included in the above five lesion groups | | | |
| ICD=International Statistical Classification of Diseases; CHD=congenital heart disease; Complex cases: lesion groups 1-2, Non-Complex cases: lesion groups 3-6 | | | | |
