# Supplementary material for: Healthcare consumption in congenital heart disease: A temporal life-course perspective following pediatric cases to adulthood
Source: Int J Cardiol Congenit Heart Dis. 2023 Jan 11;11:100440. doi: 10.1016/j.ijcchd.2023.100440 (PMC11657615; doi:10.1016/j.ijcchd.2023.100440)
Supplement: Multimedia component 4 [file mmc4.docx]

**Supplementary Table 3: Quasi Poisson Regression Results on Changes in Hospitalization Among Total CHD Population Over Time**

|  | | Infant Cases:  Age interval (0 - < 1 years) | | | Young Pediatric Cases:  Age interval (1 - < 10 years) | | | | Older Pediatric Cases:  Age interval (10 - < 18 years) | | |  |  |  |
| --- | --- | --- | --- | --- | --- | --- | --- | --- | --- | --- | --- | --- | --- | --- |
| **Birth Period** | Relative change in hospitalization | | 95% CI | Pr(>\|z\|) | Relative change in hospitalization | 95% CI | Pr(> \|z\|) | Relative change in hospitalization | | 95% CI | Pr(>\|z\|) |  |  |  |
| 1970-1974 | ***REFERENCE YEAR*** | | | | | | | | | | |  |  | ***REFERENCE YEAR*** |
| 1975-1979 | 1.23 | | [1.15, 1.31] | 0.00 | 1.16 | [1.01, 1.34] | 0.04^a^ | 1.03 | | [0.86, 1.24] | 0.75^c^ |  |  |  |
| 1980-1984 | 1.47 | | [1.39, 1.56] | 0.00 | 1.38 | [1.20, 1.58] | 0.00 | 0.92 | | [0.76, 1.10] | 0.35^c^ |  |  |  |
| 1985-1989 | 1.52 | | [1.44, 1.60] | 0.00 | 1.38 | [1.22, 1.57] | 0.00 | 0.74 | | [0.62, 0.89] | 0.00 |  |  |  |
| 1990-1994 | 1.56 | | [1.48, 1.64] | 0.00 | 1.21 | [1.07, 1.37] | 0.00 | 0.68 | | [0.58, 0.81] | 0.00 |  |  |  |
| 1995-1999 | 1.34 | | [1.27, 1.41] | 0.00 | 0.88 | [0.77, 1.00] | 0.05^a^ | 0.68 | | [0.57, 0.81] | 0.00 |  |  |  |

^a^ significant to 5%

^b^ significant to 10%

^c^ insignificant
