# Supplementary material for: Healthcare consumption in congenital heart disease: A temporal life-course perspective following pediatric cases to adulthood
Source: Int J Cardiol Congenit Heart Dis. 2023 Jan 11;11:100440. doi: 10.1016/j.ijcchd.2023.100440 (PMC11657615; doi:10.1016/j.ijcchd.2023.100440)
Supplement: Multimedia component 5 [file mmc5.docx]

**Supplementary Table 4: Quasi Poisson Regression Results on Changes in Hospitalization Among Complex CHD Cases Over Time**

|  | |  | |  |  | |  | |  |  | | |  | |  |  |  |  |  |
| --- | --- | --- | --- | --- | --- | --- | --- | --- | --- | --- | --- | --- | --- | --- | --- | --- | --- | --- | --- |
|  | | Infant Cases:  Age interval (0 - < 1 years) | | | | | Young Pediatric Cases:  Age interval (1 - < 10 years) | | | | | | Older Pediatric Cases:  Age interval (10 - < 18 years) | | | |  |  |  |
| **Birth Period** | Relative change in hospitalization | | 95% CI | | | Pr(>\|z\|) | Relative change in hospitalization | 95% CI | | | Pr(> \|z\|) | Relative change in hospitalization | | 95% CI | | Pr(>\|z\|) |  |  |  |
| 1970-1974 | ***REFERENCE YEAR*** | | | | | | | | | | | | | | | |  |  | ***REFERENCE YEAR*** |
| 1975-1979 | 1.45 | | [1.30, 1.62] | | | 0.00 | 1.14 | [0.91, 1.42] | | | 0.26^c^ | 1.30 | | [1.18, 1.42] | | 0.20^c^ |  |  |  |
| 1980-1984 | 1.82 | | [1.64, 2.03] | | | 0.00 | 1.21 | [0.97, 1.50] | | | 0.09^b^ | 1.12 | | [1.02, 1.23] | | 0.59^c^ |  |  |  |
| 1985-1989 | 2.15 | | [1.95, 2.38] | | | 0.00 | 1.37 | [1.12, 1.68] | | | 0.00 | 1.03 | | [0.94, 1.13] | | 0.87^c^ |  |  |  |
| 1990-1994 | 2.43 | | [2.21, 2.68] | | | 0.00 | 1.39 | [1.14, 1.68] | | | 0.00 | 0.99 | | [0.91, 1.08] | | 0.96^c^ |  |  |  |
| 1995-1999 | 2.58 | | [2.34, 2.84] | | | 0.00 | 1.24 | [1.00, 1.52] | | | 0.05^a^ | 1.52 | | [1.39, 1.65] | | 0.03^a^ |  |  |  |

^a^ significant to 5%

^b^ significant to 10%

^c^ insignificant
