# Supplementary material for: Healthcare consumption in congenital heart disease: A temporal life-course perspective following pediatric cases to adulthood
Source: Int J Cardiol Congenit Heart Dis. 2023 Jan 11;11:100440. doi: 10.1016/j.ijcchd.2023.100440 (PMC11657615; doi:10.1016/j.ijcchd.2023.100440)
Supplement: Multimedia component 6 [file mmc6.docx]

**Supplementary Table 5: Quasi Poisson Regression Results on Changes in Hospitalization Among Non-Complex CHD Cases Over Time**

|  | | Infant Cases:  Age interval (0 - < 1 years) | | |  | Young Pediatric Cases:  Age interval (1 - < 10 years) | | | Older Pediatric Cases:  Age interval (10 - < 18 years) | | |  |
| --- | --- | --- | --- | --- | --- | --- | --- | --- | --- | --- | --- | --- |
| **Birth Period** | Relative change in hospitalization | | 95% CI | Pr(>\|z\|) | Relative change in hospitalization | | 95% CI | Pr(>\|z\|) | Relative change in hospitalization | 95% CI | Pr(>\|z\|) |  |
| 1970-1974 | ***REFERENCE YEAR*** | | | | | | | | | | |  |
| 1975-1979 | 1.15 | | [1.07, 1.24] | 0.00 | 1.17 | | [0.98, 1.40] | 0.09^b^ | 0.93 | [0.77, 1.13] | 0.47^c^ |  |
| 1980-1984 | 1.36 | | [1.27, 1.45] | 0.00 | 1.47 | | [1.24, 1.73] | 0.00 | 0.84 | [0.69, 1.02] | 0.09^b^ |  |
| 1985-1989 | 1.36 | | [1.28, 1.45] | 0.00 | 1.44 | | [1.23, 1.69] | 0.00 | 0.66 | [0.55, 0.80] | 0.00 |  |
| 1990-1994 | 1.36 | | [1.28, 1.44] | 0.00 | 1.20 | | [1.03, 1.40] | 0.02^a^ | 0.61 | [0.51, 0.73] | 0.00 |  |
| 1995-1999 | 1.10 | | [1.03, 1.17] | 0.00 | 0.82 | | [0.69, 0.97] | 0.02^a^ | 0.51 | [0.42, 0.61] | 0.00 |  |

^a^ significant to 5%

^b^ significant to 10%

^c^ insignificant
