# Supplementary material for: Healthcare consumption in congenital heart disease: A temporal life-course perspective following pediatric cases to adulthood
Source: Int J Cardiol Congenit Heart Dis. 2023 Jan 11;11:100440. doi: 10.1016/j.ijcchd.2023.100440 (PMC11657615; doi:10.1016/j.ijcchd.2023.100440)
Supplement: Multimedia component 7 [file mmc7.docx]

**Supplementary Table 6: Total Number of Hospitalization, by Age Interval**

| Total Number (%) of Pediatric Hospitalization, by Age Interval | | | |
| --- | --- | --- | --- |
| No. of Hospitalizations | 0 - < 1 Years | 1 - < 10 Years | 10 - < 18 Years |
| 0 - 4 | 6,1879 (91.2%) | 63,306 (93.4%) | 6,6591 (98.2%) |
| 5 - 9 | 4,811 (7.1%) | 2,816 (4.2%) | 817 (1.2%) |
| 10 - 14 | 852 (1.3%) | 868 (1.3%) | 216 (0.3%) |
| 15 - 19 | 167 (0.2%) | 361 (0.5%) | 70 (0.1%) |
| 20 - 39 | 96 (0.1%) | 360 (0.5%) | 96 (0.1%) |
| ≥ 40 | 9 (0.0%) | 103 (0.2%) | 24 (0.0%) |
