# Supplementary material for: Healthcare consumption in congenital heart disease: A temporal life-course perspective following pediatric cases to adulthood
Source: Int J Cardiol Congenit Heart Dis. 2023 Jan 11;11:100440. doi: 10.1016/j.ijcchd.2023.100440 (PMC11657615; doi:10.1016/j.ijcchd.2023.100440)
Supplement: Multimedia component 8 [file mmc8.docx]

**Supplementary Table 7: Sensitivity Analysis Removing Extreme Cases of Hospitalization (i.e. >14 hospitalizations)**


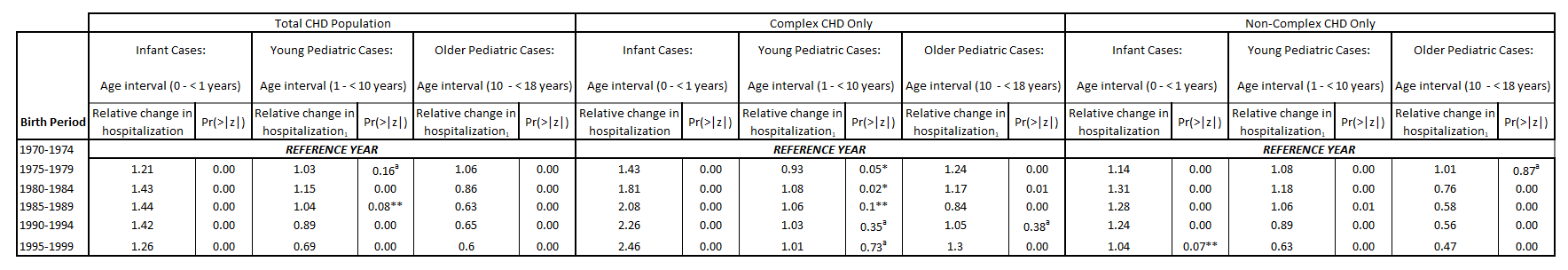
 ** significant to 5% level of significance; ** significant to 10% level of significance; ^a^ insignificant*
